# Seafood procurement auctions in the presence of origin falsification: Price-only auction versus scoring auction

**DOI:** 10.1371/journal.pone.0348582

**Published:** 2026-05-14

**Authors:** Shangjun Piao, Jiantao Guo

**Affiliations:** 1 Department of Logistics Management, School of Economics and Management, Beijing Jiaotong University, Beijing, China; 2 School of Economics and Management, Yanbian University, Yanji, Jilin, China; 3 School of Management, Zhejiang University of Technology, Hangzhou, Zhejiang, China; Universidad Complutense de Madrid, SPAIN

## Abstract

Origin falsification in seafood procurement is a significant issue that threatens supply chain integrity and distorts market competition. Despite advances in origin detection technologies, this problem persists, particularly when penalty mechanisms lack sufficient deterrent effects and fail to function effectively. In response, this study explores how different auction formats, particularly price-only and scoring auctions, can address this challenge in seafood procurement. To address this issue, we first derive the equilibrium outcomes for both price-only and scoring auctions in the presence of origin falsification. Then, we analyze how origin falsification affects the equilibrium outcomes. Finally, we conducted a comparative study of the two auction formats to explore the best format for seafood purchasers. The results suggest that when the extent of origin falsification is within a certain range, the price-only auction may outperform the scoring auction for the purchaser. This relative advantage appears more noticeable when consumers show stronger preferences for the origin. Additionally, we find that under specific conditions, purchasing seafood from low-value origins may align with purchasers’ interests. From an extended numerical study, when origin detection is available, a positive detection rate reinforces the dominance of price-only auctions in cases of severe falsification. These findings contribute to the literature on procurement auctions by highlighting the need for auction design adjustments to mitigate origin falsification in seafood procurement.

## 1. Introduction

Origin falsification is a form of seafood fraud that poses a significant threat to the integrity of the seafood supply chain and represents a pervasive issue in global seafood procurement [1]. The primary reason is that the morphological characteristics of seafood, such as sea bass, caviar, salmon, cod, crab, and eel, which are influenced by environmental variations in their place of origin, are often not easily discernible through visual inspection. This makes it challenging for purchasers to accurately verify their true origins [2]. Although advanced technologies, such as DNA testing, can reliably determine the true origin, they are restricted to sample-based analysis and cannot guarantee the accuracy of the origin for an entire batch [3]. This issue is particularly evident in processed seafood, such as processed salmon and eels, where alteration of the fish’s original form complicates the differentiation of these variations [4,5]. Chen and Garcia (2014) demonstrated that a mere 30% of the salmon labeled as ’Norwegian’ in the Chinese market was authentically sourced from Norway [6]. An investigation conducted by the Associated Press in 2018 [7] revealed instances of origin falsification by the seafood supplier “Sea to Table,” which impacted major retailers such as Kroger and Walmart. Despite the company’s assertions that its products were “wild-caught” and “locally sourced,” a significant portion of the seafood was, in fact, farmed and imported. Furthermore, numerous studies have documented cases in which unethical suppliers exploit purchasers’ preferences for products from high-value origins by substituting them with products from low-value origins, thereby benefiting from falsification [8–12]. The ramifications of origin falsification are extensive, significantly eroding consumer trust in seafood products, particularly as consumers prioritize premium-priced origins (e.g., Norwegian salmon or wild-caught seafood). When these products are misrepresented, they not only result in financial losses for purchasers, but also erode the credibility of the entire seafood supply chain. Moreover, fraudulent substitution of seafood distorts market prices, engenders unfair competition, and undermines the reputation of legitimate producers and suppliers. In sum, falsification of origin poses a threat to both the economic stability of the seafood industry and the integrity of global trade.

In response to the growing demand for competitive pricing in seafood procurement, major retailers such as Carrefour, Target, Kroger, and Walmart have increasingly purchased seafood through auctions. Currently, price-only auctions are widely adopted by purchasers because they achieve low prices through strong competition among bidders while also helping prevent corruption and favoritism [13]. However, these auctions are less effective in addressing the challenge of seafood origin falsification because the effectiveness of ex-post penalty mechanisms is significantly limited. Moreover, they focused solely on price, without considering non-price factors, such as the origin of seafood. In contrast, some studies suggest that scoring auctions, which account for both price and non-price attributes, may be more suitable for mitigating certain types of fraud by incorporating additional evaluation criteria, thereby achieving optimal information revelation from sellers [14]. In procurement environments where suppliers misreport attributes other than price, such as quality and lead time, scoring auctions are traditionally regarded as superior to price-only auctions, because they better incentivize supplier competition and reduce the winner’s information rent [15]. Nevertheless, while scoring auctions theoretically offer the potential to address fraud issues by incorporating non-price factors such as origin, their effectiveness in combating origin falsification within the seafood supply chain remains under explored. This gap underscores the need to compare the suitability of price-only and scoring auctions for seafood procurement in the presence of origin falsification. Therefore, this study aims to answer the following questions:

(i) How does origin falsification affect seafood purchasers’ optimal decisions (e.g., choice of origin, scoring rule, etc.) and utilities in both price-only auctions and scoring auctions?
(ii) Which auction format is more favorable to the purchaser in the presence of origin falsification: the price-only auction or the scoring auction?

To address these problems, we compared the price-only auction and scoring auction for procuring seafood in the presence of origin falsification. Specifically, we consider a seafood procurement auction, in which a purchaser procures seafood from multiple suppliers. Seafood has multiple origins, and the purchaser’s utility is related to the origin of the seafood and its final payment. We consider two suppliers: one that is cost-efficient (strong) and the other that is cost-inefficient (weak). The weak supplier is dishonest and has a motive for origin falsification; that is, delivering seafood from a lower origin level than the promised one. If the dishonest supplier wins the contract and origin falsification is undetected by the purchaser, the purchaser’s utility is impaired according to the satisfaction risk model [16]. We study two common auction formats for the above scenario: a price-only auction and a scoring auction. First, we consider a price-only auction and derive the equilibrium outcome. In addition, we clarify the optimal origin choices for the purchaser under different extents of origin falsification, and analyze the impact of origin falsification on the purchaser’s utility. Next, we analyze the equilibrium outcome of the scoring auction and investigate the optimal origin weight and purchaser’s utility under different degrees of origin falsification. We compare the two auction formats to determine the optimal application for seafood procurement under varying extents of origin falsification. Finally, we further extend the analysis by incorporating origin detection. Unlike previous industrial procurement auctions (e.g., Chen-Ritzo et al. [15], Huang and Xia [17], and Asker and Cantillon [18]), we introduce a satisfaction risk model to capture consumers’ preferences for seafood origin.

The remainder of this paper is structured as follows: Section 2 reviews the relevant literature and identifies the existing research gap; Section 3 describes the problem; Section 4 analyses the equilibrium outcome in the price-only auction; Section 5 analyses the equilibrium outcome in the scoring auction; Section 6 compares the two auction formats; Section 7 introduces origin detection; and Section 8 concludes the paper.

## 2. Literature review

In this study, we conduct a comparative study between price-only auctions and scoring auctions for procuring seafood in the presence of origin falsification. Three streams of literature are related to this study: seafood origin fraud prevention, procurement auctions with misrepresentation of non-price information, and comparisons between price-only and scoring auctions.

The first stream of literature related to this study focuses on seafood origin fraud prevention. To date, the most widely employed methods for origin detection in seafood include protein- and DNA-based techniques, chromatography, elemental profiling, and isotopic analysis [19]. Wallstrom et al. [11] utilized mitochondrial DNA barcoding to investigate seafood labeling inaccuracies in restaurants, groceries, and sushi bars in the greater Honolulu area. Mazzeo and Siciliano [20] suggested that proteomics could serve as a reliable and practical front line detection method for fish origin identification, offering all the desirable characteristics of an official screening detection protocol, including flexibility, reliability, and reduced analysis time. Ghidini et al. [21] studied the use of qualitative spectroscopy combined with chemometrics to accurately determine the origin of fish. Although these techniques have shown great potential for unambiguously identifying the geographic origin of fish and other seafood products [22–26], they also have drawbacks, particularly regarding long testing times and the possibility of causing irreversible damage to samples during testing [19]. These drawbacks make it difficult to implement them effectively in large-scale applications, rendering them suitable only for sampling [3,20,21,27].

Some scholars advocate the use of blockchain technology to enable real-time monitoring of the seafood supply chain, positing that the transparency, immutability, and traceability of blockchain supply chain data can address issues of data falsification, including original falsification by suppliers [28,29]. Tsolakis et al. [30] argued that integrating blockchain into supply chain network design can help prevent origin falsification in seafood products and they established principles and implementation frameworks for blockchain-centered supply chain design. However, Cao et al. [31] contended that linking physical entities to digital information is susceptible to data forgery. In summary, although technological advancements offer the possibility of addressing origin falsification, this problem persists. This study analyzes the impact of origin falsification on seafood procurement from the perspective of auction design and determines the optimal auction format to mitigate the impact of origin falsification.

The second stream of literature related to this study concerns procurement auctions with misrepresentation of non-price information. Most existing literature employs mechanisms, such as penalties and effort-based incentives, to deter potential dishonesty. Qian et al. [32], Zhang et al. [33], and Yu et al. [34] examined auction mechanisms for transportation service procurement, addressing delivery risks while also considering the misreporting of non-price attributes. They focus on utilizing incentives and penalties to ensure contract fulfillment and truthful reporting of non-price attributes. Chen et al. [35] investigated procurement auctions under project quality uncertainty, addressing the adverse selection problem arising from differences in supplier types through penalty mechanisms. Huang et al. [16] reduced post-transaction moral hazard by developing performance-related incentives tied to project delivery. They argue that such mechanisms can mitigate uncertainty related to project quality, benefit both buyers and sellers, and achieve Pareto improvements. The literature above assumes that bidders’ misrepresentation of non-price information can be observed and verified ex-post. However, in cross-border seafood supply chains, ex-post penalty mechanisms often prove ineffective because of jurisdictional complexities, international political factors, and enforcement challenges across different legal systems [36]. Therefore, this study focuses on seafood procurement auctions in which origin falsification is inherently difficult to fully prevent (given the limitations of detection technologies and supply chain opacity), analyzes its impact on the outcomes of two common auction formats (price-only auctions and scoring auctions), and explores their optimal application under such conditions.

The third stream of the literature compares price-only auctions and scoring auctions. The earliest procurement auctions considered only price as a factor and are, therefore, also referred to as price-only auctions. Procurement decisions are rarely based solely on price. Concerns about the quality of the goods or services often play a critical role in the final decision [37]. In such cases, the procurer typically designs multidimensional auctions that consider both the contract that each bidder is willing to fulfill and its associated cost [38]. Che [39] pioneered the study of multi-attribute procurement auctions. Based on the context of the Department of Defense’s weapons procurement system, he integrated price and quality attributes, and provided a qualitative argument that scoring auctions outperform price-only auctions. Asker et al. [18] studied the equilibrium bidding strategies in multi-attribute scoring auctions, where the buyer also cares about non-price attributes such as lead time, time to completion, and quality. They demonstrated that scoring auctions dominate price-only auctions with a minimum quality threshold. Chen-Ritzo et al. [15] considered three attributes of the price, quality, and lead time and compared the performance of a scoring auction mechanism with that of a price-only auction through laboratory experiments. Their findings suggest that the scoring auction outperforms the price-only auction both theoretically and experimentally. Huang et al. [17] studied procurement auctions with supplier-agent collusion, where dishonest suppliers bribe agents to inflate their evaluations, and found that in this case, the advantage of scoring auctions over price-only auctions disappears. However, it is unclear whether the above conclusions can be generalized to seafood procurement auctions. This study extends the comparison between price-only auctions and scoring auctions to the seafood procurement context in the presence of origin falsification and presents new findings.

## 3. Problem description

Consider that a purchaser (retailer) procures a certain quantity of a specified seafood species from multiple potential suppliers N (fishmongers or wholesalers) through a procurement auction. Generally, we refer to the purchaser as “she” and a supplier as “he”. In the auction, one winner wins the entire contract; therefore, the quantity is normalized to one. Usually, there are multiple origins for one species of seafood with varying qualities. We introduce a definition of “origin level” to measure a comprehensive evaluation of seafood’s non-price attributes such as freshness, flavor, quality, and specification from a given origin. Each seafood origin has a unique origin level o∈O (where O is the set of all possible origin levels) and a higher origin level provides higher value to the purchaser. For ease of analysis, we assume that O is a continuum. If the winner promises the seafood from origin o, the purchaser derives utility U(p,o)=o−p, where p is the money paid to the winner. Similar utility functions can often be found in the literature [40,41] for multi-attribute procurement auctions.

Each supplier i∈N can provide the seafood from all origins and his cost function $c\left(o,\theta_{i}\right)$ is related to origin o and his cost parameter $\theta_{i}$. We assume that cost function c(o,θ) has the following properties:

(i) c(0,θ)=0, $c_{o}=\partial c/\partial o>0$ (higher origin levels require higher costs for a supplier);
(ii) $\underset{o\to 0}{lim}c_{o}=0$, $c_{\theta }=\partial c/\partial \theta >0$ (a cost-inefficient supplier has a higher cost with a lower θ indicates higher productive efficiency, e.g., superior technology, more efficient management, or access to cheaper inputs. This ordering of types ($\theta_{\text{1}}<\theta_{\text{2}}$) ensures that the strong supplier (with lower θ) has a cost advantage at any given origin);
(iii) $c_{o\theta }=\partial^{\text{2}}c/\partial o\partial \theta >0$ (a cost-inefficient supplier has a higher marginal cost for the origin level);
(iv) $c_{oo\theta }=\partial^{\text{3}}c/\partial o^{\text{2}}\partial \theta >0$ (a cost-inefficient supplier exhibits a higher rate at which marginal cost changes with respect to origin; for example, $c=o^{\text{2}}\theta$).

Furthermore, we impose the technical assumptions $c_{oo}={\partial^{\text{2}}c/\partial o}^{\text{2}}>0$ and $c_{ooo}=\partial^{\text{3}}c/\partial o^{\text{3}}>-\overset{\text{2}}{\underset{oo}{c}}$ to ensure that there is a unique optimal origin level to maximize the purchaser’s utility function and there is a uniqueness of equilibrium in the case without origin falsification, respectively. If supplier i wins the contract with promised origin $o_{i}$ at price p, his payoff is $\pi \left(p,o_{i},\theta_{i}\right)=p-c\left(o_{i},\theta_{i}\right)$.

As mentioned in the Introduction, penalty mechanisms for origin falsification may be ineffective, giving suppliers economic incentives to engage in unethical falsification. To analyze the impact of origin falsification on seafood procurement more clearly, we consider a seafood procurement scenario in which origin falsification is unavoidable. That is, supplier i may promise seafood from origin o but provide that from another origin $o_{i}$ ($o_{i}<o$). If $o_{i}\neq o$, we say that supplier i is dishonest and denote $m=o-o_{i}>0$ as the range of origin falsification. To facilitate analysis, we assume m is exogenous, but the impact of m on the results will be discussed later. If a dishonest supplier wins the contract, the purchaser will bear a utility loss Δo due to the origin falsification. Denote Δo=m(1+ε), where ε is a consumer-specific uncertainty factor to capture consumers’ heterogeneous preferences for seafood origin. We assume that ε U[0,a], similar to the uncertainty factor of satisfaction in [16]. A higher value of ε indicates greater consumer sensitivity to seafood origin falsification, which is determined by individual factors rather than the purchaser’s desired origin value 𝑜. Therefore, ε is independent of o.

**Fig 1:**
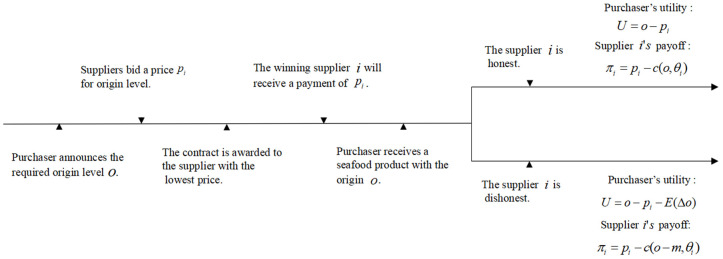
Timeline of price-only auctions [18].

**Fig 2:**
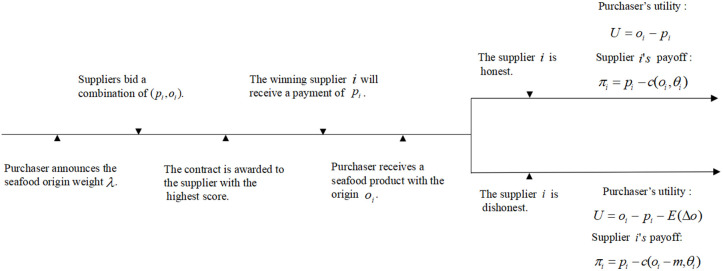
Timeline of scoring auctions [18].

We consider that the purchaser can choose one of the two common procurement auction formats: a price-only auction (P) or a scoring auction (S). Specifically, we consider the first-price and first-score auctions. Drawing upon the standardized procurement process framework developed by Asker and Cantillon [18], and incorporating the specific context of this study, the timelines of the two auction formats are shown in Figs 1 and 2.

Price-only auction: If the purchaser chooses P, she first announces the required origin level o, and then each seafood supplier i∈N bids a sealed price $p_{i}$ for the seafood from this origin o. The supplier with the lowest price wins the contract and he will get the payment as the same as he bids.

Scoring auction: If the purchaser chooses S, she will not restrict the origin but will first announce a scoring rule s(p,o)=λo−p, where λ≥0 represents the monetary equivalent of origin, indicating its weight in valuation. Then each supplier i∈N bids sealed origin level $o_{i}$ and price $p_{i}$. The supplier with the highest score wins the contract and he delivers the seafood as he promised and gets the payment as the same as he bids.

To facilitate the analysis and obtain analytical solutions, we consider a case with two suppliers i=1,2 in this paper. Without loss of generality, assume that $\theta_{\text{1}}<\theta_{\text{2}}$. Thus, supplier 1 is strong and supplier 2 is weak. We assume that if there is origin falsification, the weak supplier will always be dishonest, as weaker firms are often less trustworthy [42]. If dishonest supplier 2 misreports his origin, he will deliver the seafood from a lower origin level than the promised level and report the price according to the actual cost. The purchaser knows that one supplier is strong and honest while the other is weak and dishonest, but cannot distinguish which is which. To simplify the analysis, we adopt the tie-breaking rule, in which a strong supplier wins the contract in the event of a tie [41].

## 4. Price-only auction

If the purchaser chooses P, she firstly announces the origin of a particular seafood species as o, and then both suppliers will provide seafood from the same origin. We consider a first-price auction in which suppliers compete in a Bertrand fashion. In equilibrium of a first-price auction with complete information, the cost-effective supplier bids slightly below the opponent’s cost, while the cost-ineffective supplier bids its own cost. Given the payment rule of a first-price auction, this implies that the winning supplier receives a payment approximately equal to the opponent’s cost.

### 4.1. The case without origin falsification

In equilibrium, supplier 1 wins the contract and his payment is $p=c\left(o,\theta_{\text{2}}\right)$ because $c\left(o,\theta_{\text{1}}\right)<c\left(o,\theta_{\text{2}}\right)$. Supplier 1 can earn a payoff $\overset{P}{\underset{T}{R}}\equiv \pi_{\text{1}}=c\left(o,\theta_{\text{2}}\right)-c\left(o,\theta_{\text{1}}\right)$, which is a type of information rent and we refer to it as technological rent. Because the purchaser does not know the efficiency (type) of the suppliers, the most efficient supplier receives a rent due to incomplete information. The literature typically refers to this rent as the information rent. In this paper, because there is also incomplete information regarding origin falsification, we use the terms “technological rent” and “origin falsification rent” to distinguish between these two rents. The purchaser’s utility is $U^{P}\left(o\right)=o-c\left(o,\theta_{\text{2}}\right)$ and there is optimal origin level $o_{SB}$ to maximize her utility, i.e., $o_{SB}\equiv argmaxo-c\left(o,\theta_{\text{2}}\right)$, also referred to the second-best origin. Since $U^{P}(o{)}^{\prime }=1-c_{o}\left(o,\theta_{\text{2}}\right),\;U^{P}{\left(o\right)}^{\prime \prime }=-c_{oo}\left(o,\theta_{\text{2}}\right)<0$ and $o_{SB}$ is determined by $1-c_{o}\left(o,\theta_{\text{2}}\right)=0$. We use $\overset{P}{\underset{SB}{U}}\left(o\right)$ to represent the corresponding second-best utility.

If the purchaser has complete information about each supplier’s cost, her utility is $U^{P}\left(o\right)=o-c\left(o,\theta_{\text{1}}\right)$, which is equal to the maximum social welfare. According to our setup of the cost function, the utility has a unique maximum first-best origin, denoted as $o_{FB}\equiv argmaxo-c\left(o,\theta_{\text{1}}\right)$, also referred to as the socially effective origin level. We use $\overset{P}{\underset{FB}{U}}\left(o\right)$ to represent the corresponding first-best utility.

### 4.2. The case with origin falsification

If there is origin falsification, supplier 2 will compete by falsifying a lower origin level for the required origin level, i.e., delivering an origin level o−m instead of o. Thus, origin falsification allows weak-and-dishonest supplier 2 to meet the minimum origin level at a lower cost. We define the amount of cost savings as the origin falsification rent, i.e., $\overset{P}{\underset{O}{R}}\equiv c\left(o,\theta_{\text{2}}\right)-c(o-m,\theta_{\text{2}})$.

In equilibrium, the winning supplier bids up to the opponent’s cost. If $c(o-m,\theta_{\text{2}})\geq c\left(o,\theta_{\text{1}}\right)$ or equivalently, $\overset{P}{\underset{O}{R}}\leq \overset{P}{\underset{T}{R}}$, supplier 1 wins the contract and his payoff is $\pi_{\text{1}}=c(o-m,\theta_{\text{2}})-c\left(o,\theta_{\text{1}}\right)$. Otherwise, supplier 2 wins the contract. Therefore, supplier 2 can earn a positive profit if and only if $c(o-m,\theta_{\text{2}})<c\left(o,\theta_{\text{1}}\right)$. His payoff is $\pi_{\text{2}}=c\left(o,\theta_{\text{1}}\right)-c(o-m,\theta_{\text{2}})$.

Where E(Δo) is the expected loss of utility due to this origin falsification.

Let $\overset{P}{\underset{i}{U}}\left(o\right)$ denote the purchaser’s utility, where subscript i indicates which supplier wins the contract. If supplier 1 wins the contract, we have that


$$U^{P}\left(o\right)=\overset{P}{\underset{\text{1}}{U}}\left(o\right)=o-c(o-m,\theta_{\text{2}})$$ (1)

If supplier 2 wins the contract, we have that


$$U^{P}\left(o\right)=\overset{P}{\underset{\text{2}}{U}}\left(o\right)=o-c\left(o,\theta_{\text{1}}\right)-E(\Delta o)=o-c\left(o,\theta_{\text{1}}\right)-m(1+E(\varepsilon ))$$ (2)

Next, we proceed to compare the magnitudes of $c\left(o,\theta_{\text{1}}\right)$ and $c(o-m,\theta_{\text{2}})$. Because $c_{o\theta }>0$, c(o,θ) satisfies the single-crossing property in (o;θ) [43]. Then the single-crossing property holds between $c\left(o,\theta_{\text{1}}\right)$ and $c(o-m,\theta_{\text{2}})$ (please see Appendix A.1 for details). Denote this positive crossing point as $\hat{o}$, which makes $c(\hat{o}-m,\theta_{\text{2}})=c\left(\hat{o},\theta_{\text{1}}\right)$. Then, we obtain the following lemma.

**Lemma 1.** *If* m>0 *is continuous,*

*(I) there exists* $\hat{o}>0$ *such that*


$$\left\{ \begin{array}{c} @l@c(o-m,\theta_{\text{2}})<c\left(o,\theta_{\text{1}}\right),\;o<\hat{o}\\ c(o-m,\theta_{\text{2}})=c\left(o,\theta_{\text{1}}\right),\;o=\hat{o}\\ c(o-m,\theta_{\text{2}})>c\left(o,\theta_{\text{1}}\right),\;o>\hat{o} \end{array}\right.$$

*(II)* $\hat{o}$ *is increasing and convex with* m.

*Proof*. Please see Appendix A.1.

From **Lemma 1**, the origin level of seafood for the species selected by the purchaser affects the auction outcome. If the purchaser chooses a seafood origin level that is not lower than $\hat{o}$, honest and strong supplier 1 will win and the purchaser will not have the loss of utility due to origin falsification. However, if the purchaser chooses an origin level below $\hat{o}$, dishonest supplier 2 will win the contract. This is because for a higher origin level, the cost advantage of strong supplier 1 is stronger and can fully offset the spurious advantage of weak supplier 2 created by origin falsification. Therefore, to avoid origin falsification, the purchaser should choose to procure seafood from higher origin levels in price-only auctions.

**Proposition 1** shows optimal origin $o^{*}$ such to maximize the purchaser’s utility.

**Proposition 1.** *There exist two critical values for the scope of origin falsification* $\overset{P}{\underset{\text{1}}{m}}$ *and* $\overset{P}{\underset{\text{2}}{m}}$ *such that,*

*(I) If* $\;E(\varepsilon )\geq \frac{o_{2}-c(o_{2},\theta_{1})-\left(\hat{o}-c(\hat{o}-m,\theta_{2})\right)}{m}-1,$


$$o^{*}=\left\{ \begin{array}{c} @c@o_{\text{1}}\equiv \text{argmax}\overset{P}{\underset{\text{1}}{U}}\left(o\right)\;\;m\leq \overset{P}{\underset{\text{1}}{m}}\\ \hat{o}\;\;\overset{P}{\underset{\text{1}}{m}}<m \end{array}\right.$$ (3)

*(II) If* $E(\varepsilon )<\frac{o_{2}-c(o_{2},\theta_{1})-(\hat{o}-c(\hat{o}-m,\theta_{1}))}{m}-1,$


$$o^{*}=\left\{ \begin{array}{c} @l@o_{\text{1}}\equiv \text{argmax}\overset{P}{\underset{\text{1}}{U}}\left(o\right)\;\;m\leq \overset{P}{\underset{\text{1}}{m}}\\ \hat{o}\;\;\overset{P}{\underset{\text{1}}{m}}<m\leq \overset{P}{\underset{\text{2}}{m}}\\ o_{\text{2}}\equiv \text{argmax}\overset{P}{\underset{\text{2}}{U}}\left(o\right)\;\;\overset{P}{\underset{\text{2}}{m}}<m \end{array}\right.$$ (4)

*Proof.* Please see Appendix A.2.

From **Proposition 1**, we know that the purchaser should consider the extent of origin falsification from dishonest suppliers when determining the optimal origin level in the price-only auction.

If the expectation of consumers’ random preferences for the seafood origin is large enough ($E(\varepsilon )\geq \frac{o_{2}-c(o_{2},\theta_{1})-(\hat{o}-c(\hat{o}-m,\theta_{2}))}{m}-1$), supplier 2 winning the contract always provides less utility than supplier 1 to the purchaser. Thus, the purchaser’s optimal choice is to make supplier 1 win the contract and select $o^{*}$ to maximize her utility. There exists a critical $\overset{P}{\underset{\text{1}}{m}}$ such that $o_{\text{1}}=\hat{o}\left(\overset{P}{\underset{\text{1}}{m}}\right)$. Therefore, the purchaser should select origin level $o_{\text{1}}$ for $m\leq \overset{P}{\underset{\text{1}}{m}}$, and select origin level $\hat{o}$ for $m>\overset{P}{\underset{\text{1}}{m}}$ to maximize her utility.

Next, we consider the case where the expectation of consumers’ random preferences for the seafood origin is not too large ($E(\varepsilon )<\frac{o_{2}-c(o_{2},\theta_{1})-(\hat{o}-c(\hat{o}-m,\theta_{2}))}{m}-1$). In this case, strong supplier 1’s cost advantage will not always dominate the false advantage of weak dishonest supplier 2 from the origin falsification. Thus, supplier 1 will not always be superior to supplier 2 for the purchaser. Therefore, there exists a critical $\overset{P}{\underset{\text{2}}{m}}$ such that $\overset{P}{\underset{\text{2}}{U}}\left(o_{\text{2}}\right)=\overset{P}{\underset{\text{1}}{U}}\left(\hat{o}\left(\overset{P}{\underset{\text{2}}{m}}\right)\right)$. When $m\leq \overset{P}{\underset{\text{2}}{m}}$, the purchaser will tend to let supplier 1 win, in a situation similar to **Proposition 1(I)**. When the extent of origin falsification is small ($m\leq \overset{P}{\underset{\text{1}}{m}}$), the purchaser should select origin level $o_{\text{1}}$; for $\overset{P}{\underset{\text{1}}{m}}<m\leq \overset{P}{\underset{\text{2}}{m}}$, the purchaser should select origin $\hat{o}$ to maximize her utility. However, when $m>\overset{P}{\underset{\text{2}}{m}}$, the purchaser should select an origin level to ensure that dishonest supplier 2 can win. This is contrary to our intuition that a purchaser should procure seafood from an honest supplier. This is because when origin falsification is unavoidable and it is sufficiently large ($m>\overset{P}{\underset{\text{2}}{m}}$), if the purchaser wants supplier 1 to win, she needs to choose a sufficiently large origin level that deviates even more from the optimal one, leading to a worse decrease in the purchaser’s utility even than the origin falsification. Thus, the optimal origin choice for the purchaser is to allow supplier 2 to win and meanwhile maximize her utility, i.e., $o_{\text{2}}$.

To see the impact of the degree of origin falsification m on the purchaser’s utility, we conduct a numerical experiment. Detailed data are provided in S1 File. We set $c=o^{\text{2}}\theta_{i}$, $\theta_{\text{1}}=1$, $\theta_{\text{2}}=1.3$. Based on this cost function, the falsification rent can be expressed explicitly, i.e., $\overset{P}{\underset{O}{R}}=(2o-m)m\theta$. The condition $\partial \overset{P}{\underset{O}{R}}/\partial m>0$ implies that the more the supplier falsifies, the greater the falsification rent he obtains. Similarly, $\partial \overset{P}{\underset{O}{R}}/\partial \theta >0$ indicates that the less efficient supplier (i.e., the higher his costs), the greater the relative benefit he gains from falsification. Finally, $\partial \overset{P}{\underset{O}{R}}/\partial o>0$ shows that the higher the origin value desired by the purchaser, the greater the premium the supplier can capture through misrepresentation. We further consider two cases regarding consumers’ random preferences for the seafood origin: (i) a low-level consumer’s preference expectation (E(ε)=0), and (ii) a high-level preference expectation (E(ε)=2). For example, oysters belong to the former. Nguyen et al. [44] conducted a study on the quality attributes of seafood in the French market and found that consumers are indifferent to the origin of certain species, such as oysters, but place greater emphasis on the freshness of the seafood products. The Italian sea-bass is an example of the latter. Cantillo et al.’s [45] analysis of consumers’ preferences found that Italian consumers exhibit a particularly strong preference for sea-bass produced in Italy.

**Fig 3:**
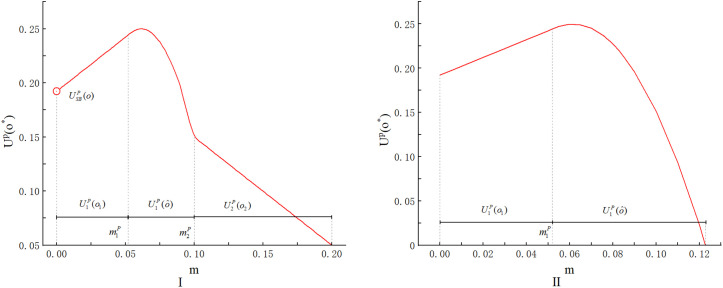
The trend of the purchaser’s optimal utility as m changes.

Fig 3(I) illustrates the trend of the purchaser’s optimal utility as m changes in E(ε)=0 scenario.

From Fig 3(I), we find that when m is relatively small, the purchaser’s optimal utility increases with m. This is because preventing misreporting requires setting a higher origin than the second-best level, which moves closer to the first-best level, and thus leads to an increase in the purchaser’s utility. In addition, we can find that the purchaser can achieve a better outcome compared to the case without origin falsification (i.e., $\overset{P}{\underset{\text{1}}{U}}\left(o_{\text{1}}\right)>\overset{P}{\underset{SB}{U}}\left(o\right)$) when m is smaller. From Equation(1), we have that $\overset{P}{\underset{\text{1}}{U}}\left(o\right)=o-c\left(o,\theta_{\text{1}}\right)-\overset{P}{\underset{T}{R}}+\overset{P}{\underset{o}{R}}=o-c\left(o,\theta_{\text{2}}\right)+\overset{P}{\underset{o}{R}}>o-c\left(o,\theta_{\text{2}}\right)=\overset{P}{\underset{SB}{U}}\left(o\right)$. We can also say this phenomenon is attributed to the origin falsification rent partially offsetting the technological rent. As m grows larger, deterring origin falsification requires the purchaser to incentivize a higher origin level that eventually exceeds the first-best level. Compensating for such an elevated origin becomes increasingly costly. Thus, the purchaser’s utility decreases. When $m\geq \overset{P}{\underset{\text{2}}{m}}$, the purchaser lets supplier 2 win and has to bear the utility loss due to the origin falsification, so her utility still decreases with m.

Fig 3(II) illustrates the trend of the purchaser’s optimal utility as m changes in the case (E(ε)=2). When consumers are very concerned about the origin of seafood, the purchaser’s utility in Fig 3(II) also exhibits an initial increase followed by a subsequent decrease as m increases. Compared to the case with E(ε)=0, the key difference is that the auction winner is consistently a strong and honest supplier rather than a weak and dishonest supplier, even m exceeds the certain threshold $\overset{P}{\underset{\text{2}}{m}}$. This phenomenon can be explained as follows. When consumers’ preference for the origin of seafood is very high, even though selecting a sufficiently large origin to prevent origin falsification is prohibitively expensive, the utility loss resulting from failing to meet consumers’ preferences is more critical. Therefore, the purchaser chooses the honest supplier to prevent consumer attrition.

## 5. Scoring auction

When the purchaser chooses S, she will first announce the scoring rule s(p,o)=λo−p. For origin weight λ, the purchaser has a freedom to overstate (λ>1) or understate (λ<1). After the purchaser announces the scoring rule, the suppliers select the optimal origin that maximizes their score. As we know from Burguet [41], Asker and Cantillon [18], for any given λ, it is a weakly dominant strategy for supplier i to choose origin $o_{i}\left(\lambda \right)\equiv {argmax}_{o}\left\{\lambda o-c\left(o,\theta_{i}\right)\right\}$. We refer to $\lambda o_{i}\left(\lambda \right)-c\left(o_{i}\left(\lambda \right),\theta_{i}\right)={\overset{―}{s}}_{i}$ as the highest score that supplier i can obtain [17]. In equilibrium, the supplier with a higher maximum score wins the contract by sightly outbidding the other supplier.

### 5.1. The case without origin falsification

When there is no origin falsification (m=0), c(o,θ) satisfies the single-crossing property [43], which means that supplier 2 has a higher marginal cost than supplier 1. Together with strict convexity of c(o,θ), this property implies that for any λ≥0, $o_{\text{1}}\left(\lambda \right)>o_{\text{2}}\left(\lambda \right)$ [41]. In equilibrium, supplier 1 wins the contract (because $d{\overset{―}{s}}_{i}/d\theta <0$) by proposing the price $p_{\text{1}}$ such that $s_{\text{1}}=\lambda o_{\text{1}}\left(\lambda \right)-p_{\text{1}}={\overset{―}{s}}_{\text{2}}=\lambda o_{\text{2}}\left(\lambda \right)-c\left(o_{\text{2}}\left(\lambda \right),\theta_{\text{2}}\right)$. Therefore,

$p_{\text{1}}=\lambda o_{\text{1}}\left(\lambda \right)-{\overset{―}{s}}_{\text{2}}\left(\lambda \right)=\lambda o_{\text{1}}\left(\lambda \right)-\lambda o_{\text{2}}\left(\lambda \right)+c\left(o_{\text{2}}\left(\lambda \right),\theta_{\text{2}}\right)$, and supplier 1 obtains payoff $\overset{S}{\underset{T}{R}}\equiv p_{\text{1}}-c\left(o_{\text{1}}\left(\lambda \right),\theta_{\text{1}}\right)=\lambda o_{\text{1}}\left(\lambda \right)-c\left(o_{\text{1}}\left(\lambda \right),\theta_{\text{1}}\right)-\left(\lambda o_{\text{2}}\left(\lambda )-c(o_{\text{2}}\left(\lambda \right),\theta_{\text{2}}\right)\right)$, which is the technological rent under S.

In equilibrium, the purchaser’s utility is $\overset{S}{\underset{SB}{U}}\left(\lambda \right)=o_{\text{1}}\left(\lambda \right)-\left(c\left(o_{\text{1}}\left(\lambda \right),\theta_{\text{1}}\right)+\overset{S}{\underset{T}{R}}\right)$ and there is a unique second-best origin weight, denoted as $\lambda_{SB}<1$ to maximize her utility. Similar to the price-only auction, if the purchaser has complete information about each supplier’s cost, she would procure from supplier 1 and choose $\lambda_{FB}={max}_{\lambda }\left\{o_{\text{1}}\left(\lambda )-c(o_{\text{1}}\left(\lambda \right),\theta_{\text{1}}\right)\right\}=1$. **Lemma 2** shows the values of $\lambda_{SB}$ and $\lambda_{FB}$.

**Lemma 2**. *If* λ>0 *is continuous,*

*(I) there is a unique* $\lambda_{SB}\equiv {argmax}_{\lambda \in [0,\infty )}\overset{S}{\underset{SB}{U}}\left(\lambda \right)<1$;
*(II)* $\lambda_{FB}=1$.

*Proof.* Please see Appendix A.3.

**Lemma 2** demonstrates that under complete information, the purchaser can achieve the first-best outcome without the need to account for rent extraction ($\lambda_{FB}=1$). However, in the presence of information asymmetry, the purchaser opts to distort the origin weight ($\lambda_{SB}<1$) as a strategic measure to curtail supplier 1’s rent, albeit at the cost of diminished origin efficiency.

### 5.2. The case with origin falsification

If supplier 2 falsifies the origin, it will misreport the origin as $o_{\text{2}}\left(\lambda \right)+m$ but deliver $o_{\text{2}}\left(\lambda \right)$, because ${\tilde{o}}_{\text{2}}\left(\lambda \right)\equiv {argmax}_{o}\left\{\lambda o-c\left(o-m,\theta_{\text{2}}\right)\right\}=o_{\text{2}}\left(\lambda \right)+m$, which maximizes its score. Then, the maximum score that supplier 2 can obtained is ${\overset{―}{s}}_{\text{2}}\left(\lambda \right)=\lambda o_{\text{2}}\left(\lambda \right)-c\left(o_{\text{2}}\left(\lambda \right),\theta_{\text{2}}\right)+\lambda m$. We define $\overset{S}{\underset{O}{R}}\equiv \lambda m$ as the origin falsification rent of supplier 2. **Lemma 3** shows that the auction outcome is closely tied to the purchaser’s origin weight.

**Lemma 3.** *If* m>0 *is continuous,*

*(I) there exists* $\hat{\lambda }>0$ *such that*


$$\left\{ \begin{array}{c} @l@{\overset{―}{s}}_{\text{1}}\left(\lambda \right)<{\overset{―}{s}}_{\text{2}}\left(\lambda \right),\;\lambda <\hat{\lambda }\\ {\overset{―}{s}}_{\text{1}}\left(\lambda \right)={\overset{―}{s}}_{\text{2}}\left(\lambda \right),\;\lambda =\hat{\lambda }\\ {\overset{―}{s}}_{\text{1}}\left(\lambda \right)>{\overset{―}{s}}_{\text{2}}\left(\lambda \right),\;\lambda >\hat{\lambda } \end{array}\right.$$

*(II)* $\hat{\lambda }$ *is increasing and convex with* m.

*Proof*. Please see Appendix A.4.

From **Lemma 3**, the origin weight of the seafood selected by the purchaser affects the auction outcome. Similar to the price-only auction, if the purchaser chooses λ that is not lower than $\hat{\lambda }$, honest and strong supplier 1 will win the contract in equilibrium. However, if the purchaser chooses λ that is below $\hat{\lambda }$, dishonest supplier 2 will win the contract. Therefore, the purchaser should choose a higher weight for the origin to avoid origin falsification in scoring auctions.

Let $\overset{S}{\underset{i}{U}}\left(\lambda \right)$ denote the purchaser’s utility, where the subscript i indicates which supplier wins the contract. If supplier 1 wins the contract, $p_{\text{1}}=\lambda o_{\text{1}}\left(\lambda \right)-{\overset{―}{s}}_{\text{2}}\left(\lambda \right)$. We have that


$$U^{S}\left(\lambda \right)=\overset{S}{\underset{\text{1}}{U}}\left(\lambda \right)=o_{\text{1}}\left(\lambda \right)-p_{\text{1}}=o_{\text{1}}\left(\lambda \right)-c\left(o_{\text{1}}\left(\lambda \right),\theta_{\text{1}}\right)-\overset{S}{\underset{T}{R}}+\overset{S}{\underset{O}{R}}$$ (5)

If supplier 2 wins the contract, $p_{\text{2}}=\lambda o_{\text{2}}\left(\lambda \right)+\lambda m-{\overset{―}{s}}_{\text{1}}\left(\lambda \right)$. We have that


$$U^{S}\left(\lambda \right)=\overset{S}{\underset{\text{2}}{U}}\left(\lambda \right)=o_{\text{2}}\left(\lambda \right)-p_{\text{2}}-\lambda mE\left(\varepsilon \right)=o_{\text{2}}\left(\lambda \right)-c\left(o_{\text{2}}\left(\lambda \right),\theta_{\text{2}}\right)+\overset{S}{\underset{T}{R}}-\overset{S}{\underset{O}{R}}-\lambda mE\left(\varepsilon \right)$$ (6)

**Proposition 2** shows optimal origin weight $\lambda^{*}$ such to maximize the purchaser’s utility.

**Proposition 2.** *There exist two critical values for the scope of origin falsification* $\overset{S}{\underset{\text{1}}{m}}$, $\overset{S}{\underset{\text{2}}{m}}$*, such that,*

*(I) If* $E\left(\varepsilon \right)\geq \frac{(1-\lambda_{\text{2}})o_{\text{2}}\left(\lambda_{\text{2}}\right)+\lambda_{\text{2}}o_{\text{1}}\left(\lambda_{\text{2}}\right)-o_{\text{1}}\left(\hat{\lambda }\right)+c\left(o_{\text{1}}\left(\hat{\lambda }\right),\theta_{\text{1}}\right)-c\left(o_{\text{1}}\left(\lambda_{\text{2}}\right),\theta_{\text{1}}\right)}{\lambda_{\text{2}}m}-1$


$$\lambda^{*}=\left\{ \begin{array}{c} @l@\lambda_{\text{1}}\equiv \text{argmax}\overset{S}{\underset{\text{1}}{U}}\left(\lambda \right)\text{ for}m\leq \overset{S}{\underset{\text{1}}{m}}\\ \hat{\lambda }\text{ for}\overset{S}{\underset{\text{1}}{m}}<m \end{array}\right.$$ (7)

*(II) If* $E\left(\varepsilon \right)<\frac{(1-\lambda_{\text{2}})o_{\text{2}}\left(\lambda_{\text{2}}\right)+\lambda_{\text{2}}o_{\text{1}}\left(\lambda_{\text{2}}\right)-o_{\text{1}}\left(\hat{\lambda }\right)+c\left(o_{\text{1}}\left(\hat{\lambda }\right),\theta_{\text{1}}\right)-c\left(o_{\text{1}}\left(\lambda_{\text{2}}\right),\theta_{\text{1}}\right)}{\lambda_{\text{2}}m}-1$


$$\begin{array}{c} \lambda^{*}=\left\{ \begin{array}{c} @l@\lambda_{\text{1}}\equiv \text{argmax}\overset{S}{\underset{\text{1}}{U}}\left(\lambda \right)\;\;\text{for}\;m\leq \overset{S}{\underset{\text{1}}{m}}\\ \hat{\lambda }\;\;\;\;\;\;\;\text{for}\;\overset{S}{\underset{\text{1}}{m}}<m\leq \overset{S}{\underset{\text{2}}{m}}\\ \lambda_{\text{2}}\equiv \text{argmax}\overset{S}{\underset{\text{2}}{U}}\left(\lambda \right)\;\;\text{for}\;\overset{S}{\underset{\text{2}}{m}}<m \end{array}\right. \end{array}$$ (8)

*Proof.* Please see Appendix A.5.

Similar to the price-only auction**, Proposition 2** indicates that the purchaser should account for the degree of origin falsification by dishonest suppliers when determining the optimal origin weight in the scoring auction. If consumers’ preferences for seafood origin are sufficiently strong ($E\left(\varepsilon \right)\geq \frac{(1-\lambda_{\text{2}})o_{\text{2}}\left(\lambda_{\text{2}}\right)+\lambda_{\text{2}}o_{\text{1}}\left(\lambda_{\text{2}}\right)-o_{\text{1}}\left(\hat{\lambda }\right)+c\left(o_{\text{1}}\left(\hat{\lambda }\right),\theta_{\text{1}}\right)-c\left(o_{\text{1}}\left(\lambda_{\text{2}}\right),\theta_{\text{1}}\right)}{\lambda_{\text{2}}m}-1$), then awarding the contract to supplier 2 always results in lower utility for the purchaser compared to supplier 1. Consequently, the purchaser’s optimal strategy is to ensure supplier 1 wins the contract and to select $\lambda^{*}$ to maximize utility. There exists a threshold value $\overset{S}{\underset{\text{1}}{m}}$ such that $\lambda_{\text{1}}=\hat{\lambda }\left(\overset{S}{\underset{\text{1}}{m}}\right)$. Hence, for $m\leq \overset{S}{\underset{\text{1}}{m}}$, the purchaser should adopt the origin weight $\lambda_{\text{1}}$, whereas for $m>\overset{S}{\underset{\text{1}}{m}}$, the optimal choice is $\hat{\lambda }$.

Next, we examine the scenario where consumers’ preferences for seafood origin are moderate ($E\left(\varepsilon \right)<\frac{(1-\lambda_{\text{2}})o_{\text{2}}\left(\lambda_{\text{2}}\right)+\lambda_{\text{2}}o_{\text{1}}\left(\lambda_{\text{2}}\right)-o_{\text{1}}\left(\hat{\lambda }\right)+c\left(o_{\text{1}}\left(\hat{\lambda }\right),\theta_{\text{1}}\right)-c\left(o_{\text{1}}\left(\lambda_{\text{2}}\right),\theta_{\text{1}}\right)}{\lambda_{\text{2}}m}-1$). In this case, the cost advantage of strong supplier 1 does not always outweigh the deceptive benefit dishonest supplier 2 gains from origin falsification, meaning that supplier 1 is not consistently superior to supplier 2 from the purchaser’s perspective. Accordingly, there exists a critical threshold $\overset{S}{\underset{\text{2}}{m}}$ such that $\overset{S}{\underset{\text{2}}{U}}\left(\lambda_{\text{2}}\right)=\overset{S}{\underset{\text{1}}{U}}\left(\hat{\lambda }\left(\overset{S}{\underset{\text{2}}{m}}\right)\right)$. For $m\leq \overset{S}{\underset{\text{2}}{m}}$, the purchaser is inclined to favor supplier 1, aligning with the scenario in **Proposition 2**(I). When origin falsification is minimal ($m\leq \overset{S}{\underset{\text{1}}{m}}$), the purchaser should set the origin weight at $\lambda_{\text{1}}$. For intermediate levels of falsification $\overset{S}{\underset{\text{1}}{m}}<m\leq \overset{S}{\underset{\text{2}}{m}}$, selecting $\hat{\lambda }$ is optimal. However, when falsification is extensive $m>\overset{S}{\underset{\text{2}}{m}}$, the purchaser’s best response is to structure the scoring auction in a way that enables supplier 2 to win. Although counter intuitive (since an honest supplier would typically be preferred) this occurs because preventing supplier 2’s victory would require inflating the origin weight excessively, leading to substantial utility losses. Overemphasizing the origin weight within the scoring mechanism ultimately forces the purchaser to pay an unjustifiably high cost for origin compliance. Therefore, the purchaser’s optimal decision is to allow supplier 2 to win while maximizing utility, i.e., $\lambda_{\text{2}}$.

**Fig 4:**
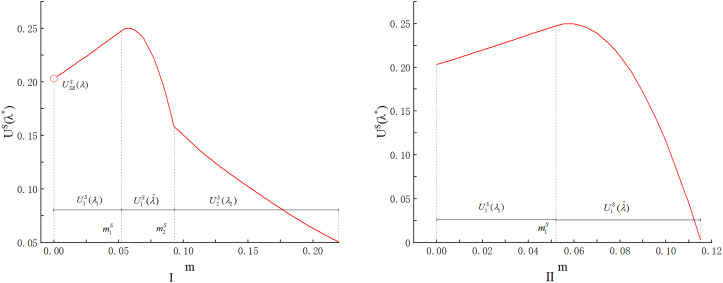
The trend of the purchaser’s optimal utility as m changes.

Next, we conduct a numerical experiment using the same parameters as in Section 4, with the results shown in Fig 4, to further investigate the relationship between the purchaser’s utility and the extent of origin falsification in the scoring auction. Fig 4(I) illustrates the trend of the purchaser’s optimal utility as m changes in E(ε)=0 scenario.

From Fig 4(I), we find that when m is relatively small, the purchaser’s optimal utility increases with m. This occurs because preventing misreporting requires setting a higher origin weight than the second-best level. As a result, the origin weight moves closer to the first-best level and thus leads to an increase in the purchaser’s utility. Moreover, it is evident that the purchaser can obtain more favorable results compared to scenarios without origin falsification. (i.e., $\overset{S}{\underset{\text{1}}{U}}\left(\lambda_{\text{1}}\right)>\overset{S}{\underset{SB}{U}}\left(\lambda_{SB}\right)$). From Equation (5), we have that $\overset{S}{\underset{\text{1}}{U}}\left(\lambda \right)=o_{\text{1}}\left(\lambda \right)-c\left(o_{\text{1}}\left(\lambda \right),\theta_{\text{1}}\right)-\overset{S}{\underset{T}{R}}+\overset{S}{\underset{o}{R}}=\overset{S}{\underset{SB}{U}}\left(\lambda \right)+\overset{S}{\underset{o}{R}}$. We can also say this phenomenon is attributed to the origin falsification rent partially offsetting the technological rent. As m grows larger, deterring origin falsification requires the purchaser to incentivize a higher origin weight that eventually exceeds the first-best level. Compensating for such an elevated origin weight becomes increasingly costly. Thus, the purchaser’s utility decreases. When $m\geq \overset{S}{\underset{\text{2}}{m}}$, the purchaser lets supplier 2 win and has to bear the utility loss due to the origin falsification, so her utility still decreases with m.

Fig 4(II) illustrates the trend of the purchaser’s optimal utility as m changes in the case (E(ε)=2). As shown in Fig 4(II), when consumers place significant importance on seafood origin, the purchaser’s utility initially rises but then declines as m increases. Compared to the case with E(ε)=0, the key difference is that the auction winner is consistently a strong and honest supplier rather than a weak and dishonest supplier, even m exceeds the certain threshold $\overset{S}{\underset{\text{2}}{m}}$. This phenomenon can be explained as follows. When consumers’ preference for seafood origin is very high, the cost of losing customer satisfaction outweighs the expense of preventing origin falsification. Thus, the purchaser chooses the honest supplier to retain the consumers.

**Proposition 1** and **Proposition 2** drive some managerial insight for management.

First, it is important to understand the nature and scope of origin falsification when designing seafood procurement auctions. Raising the minimum origin standard (or origin weight) can prevent the origin falsification supplier from winning and avoid procurement of inferior seafood.

Second, when the issue of origin falsification cannot be avoided, purchasers need to consider consumer sensitivity to the origin of seafood when developing procurement auction rules. Consumers are generally highly sensitive to the place of origin when purchasing seafood, as origin is often associated with quality, safety, and sustainability [46,47]. Studies have shown that origin labeling can significantly influence consumers’ preferences, and misleading origin claims may lead to reduced consumer trust and market inefficiencies [48,49]. Given this strong sensitivity, preventing origin falsification is crucial for purchasers. However, not all consumers prioritize their origins in their purchasing decisions. For example, Vanhonacker [50] and Witter et al. [51] found that origin is not a decisive factor among the various elements consumers consider when buying seafood. Similarly, Onozaka et al. [52] observed that a significant portion of consumers prefer domestically farmed salmon over branded, imported Norwegian salmon, indicating that the origin factor is not a priority for consumers in some cases. Thus, purchasing seafood from a lower-level of origin is not a bad choice.

Third, for consumer groups that are less concerned about the origin of seafood, purchasers must exercise greater vigilance. If strong suppliers engage in origin falsification, the impact on the purchaser’s utility could be severe. In such cases, beyond reducing technological rent by increasing the number of bidders, it is essential to implement punitive measures or regulatory mechanisms. A practical solution is to enhance peer monitoring systems, as competitors often have deeper market insights than suppliers and regulators do. By providing economic incentives for firms to report fraudulent activities, peer oversight can serve as an effective deterrent to origin falsification. Saak [53] and Fonzo [54] suggested that peer monitoring may be a more reliable approach to maintaining origin integrity than relying solely on mandatory regulations and controls.

## 6. Price-only auction versus scoring auction

**Fig 5:**
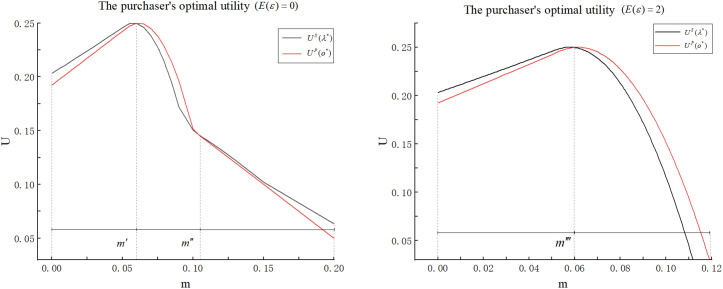
Comparison of purchasers’ optimal utility under the two auction formats.

In this section, we analyze which auction format, the price-only auction or the scoring auction, is more suitable for the purchaser to procure seafood in the presence of origin falsification. While some current studies (e.g., [18] and [15]) demonstrated that scoring auctions dominate price-only auctions for the purchaser when multiple attributes such as price, quality, and lead time are considered, they do not account for origin falsification. We compare the numerical results in Sections 4 and 5, as shown in Fig 5.

From Fig 5, we find that when consumers’ preferences level is low (E(ε)=0), the scoring auction dominates the price-only auctions for the purchaser if origin falsification remains within a small range ($m\in \left(0,m^{\prime }\right)$). This result is similar to the conclusions of Asker et al. [18]. When the extent of origin falsification m is small, S enable multidimensional competition and simplify supplier types through the use of maximum scores. Even if the scoring rule does not perfectly reflect the purchaser’s true preferences, the mechanism can still approximate optimal outcomes by adjusting the attribute (origin) weight [39]. So S can achieve higher allocation efficiency and purchaser utility than P.

However, when the extent of origin falsification falls within an intermediate range ($m\in \left(m^{\prime },m^{\prime \prime }\right)$), S is instead dominated by P. This is in contrast with previous studies (e.g., [15] and [18]). This result indicates that, within this range of m, the optimal purchaser’s utility is higher when choosing P compared to S. This is because preventing origin falsification can be costly in our study. As demonstrated in **Lemma 1** and **Lemma 3**, the purchaser must procure seafood with unnecessarily high origin levels to deter suppliers from falsifying origin information.

Under S, suppliers have full flexibility in choosing its price-origin combination, while suppliers lose flexibility in selecting the origin level under P. However, under P, the purchaser can more effectively deter origin falsification and procure seafood at a lower origin level compared to S. Specifically, under S, the purchaser must procure seafood at a high-value origin level $o_{\text{1}}\left(\hat{\lambda }\right)$ from supplier 1; Otherwise, supplier 2 can outbid supplier 1 by delivering seafood with falsified origin. Under P, however, if the purchaser imposes a strict origin requirement $o=o_{\text{1}}\left(\hat{\lambda }\right)$, supplier 2 can no longer opt for $o_{\text{2}}\left(\hat{\lambda }\right)$. This inflexibility weakens supplier 2, allowing the purchaser to enforce a lower origin standard under P compared to S, i.e., setting $o:=\hat{o}<o_{\text{1}}\left(\hat{\lambda }\right)$.

When the extent of origin falsification exceeds $m^{\prime \prime }$, S once again dominates P. This is because, at this stage, the purchaser will allow supplier 2 to win, as the cost of preventing falsification is prohibitively high under both S and P, making it more beneficial for the purchaser if supplier 2 wins. Furthermore, under the same condition where the purchaser allows supplier 2 to win, S once again demonstrates its advantage—achieving higher allocation efficiency and greater purchaser utility than P.

When consumers’ preferences level is high (E(ε)=2), S dominates P for the purchaser, provided that origin falsification remains within a small range ($m\in \left(0,m^{\prime \prime \prime }\right)$). However, unlike the case with E(ε)=0, P consistently outperforms S for all $m\in (m^{\prime \prime \prime },\infty )$. This is because, as previously discussed, although selecting a sufficiently large origin to prevent falsification is prohibitively costly, the utility loss from failing to meet consumers’ preferences becomes more significant. Therefore, under the same condition where the purchaser allows supplier 1 to win, the advantage of the P persists.

To effectively apply this finding in practice, seafood purchasers should have a clear understanding of the extent of origin falsification (m) and the expected value of consumers’ random preferences (E(ε)). On the one hand, when purchasers choose between auction formats S and P, they need to assess the extent of origin falsification. On the other hand, it is also essential to accurately understand the strength of consumers’ preferences regarding the origin of seafood products. Therefore, the practical utility of these results depends heavily on a designer’s expertise and familiarity with the procurement context.

## 7. Extension and discussion

In the previous sections, we assumed that the purchaser is unable to detect supplier origin falsification. In this section, we introduce partial detect ability, meaning that the purchaser conducts origin testing on the seafood delivered by suppliers and faces a certain probability of detecting dishonest suppliers’ falsification. We define δ as the probability that the purchaser detects origin falsification (the detection rate). We assume δ is increasing in the level of origin falsification m (i.e., dδ/dm>0) but treat its specific value as exogenously given in the sense that this relationship is technologically determined and cannot be altered by the firm in the short run.

First, we consider the price-only auction. In equilibrium, if supplier 1 wins the contract, his payoff is still $\pi_{\text{1}}=c(o-m,\theta_{\text{2}})-c\left(o,\theta_{\text{1}}\right)$. If supplier 2 wins the contract, his payoff is $c\left(o,\theta_{\text{1}}\right)-c(o-m,\theta_{\text{2}})$ if undetected, and 0 if detected. In the latter case, the purchaser will procure from an alternative channel at a price no lower than the quoted price of supplier 1. Hence, the purchaser’s utility when falsification is detected is $o-c\left(o,\theta_{\text{2}}\right)$. Then, the purchaser’s expected utility can be written as:

$E\left(\overset{P}{\underset{\text{2}}{U}}\left(o\right)\right)=(1-\delta )\cdot \left(\text{utility without detection}\right)+\delta \cdot \left(\text{utility with detection}\right)=(1-\delta )\left(o-c(o,\theta_{\text{1}})-E\left(\Delta o\right)\right)+\delta \left(o-c\left(o,\theta_{\text{2}}\right)\right)$. Supplier 2’s expected payoff is $E\left(\pi_{\text{2}}\right)=(1-\delta )\left(c\left(o,\theta_{\text{1}})-c(o-m,\theta_{\text{2}}\right)\right)$.

Then, if supplier 1 wins the contract, we have that


$$\overset{P}{\underset{\text{1}}{U}}\left(o\right)=o-c(o-m,\theta_{\text{2}})$$ (9)

If supplier 2 wins the contract, we have that


$$E\left(\overset{P}{\underset{\text{2}}{U}}\left(o\right)\right)=(1-\delta )\left(o-c(o,\theta_{\text{1}})-E\left(\Delta o\right)\right)+\delta \left(o-c\left(o,\theta_{\text{2}}\right)\right)$$ (10)

By comparing the values of equations (9) and (10), we check whether **Proposition 1(II)** still holds. When E(ε) is small (for simplicity, we set E(ε)=0), equation (10) can be written as: $E\left(\overset{P}{\underset{\text{2}}{U}}\left(o\right)\right)=(1-\delta )(o-c(o,\theta_{\text{1}})-m)+\delta \left(o-c\left(o,\theta_{\text{2}}\right)\right)$. Because dδ/dm>0, $E\left(\overset{P}{\underset{\text{2}}{U}}\left(o\right)\right)\to \delta \left(o-c\left(o,\theta_{\text{2}}\right)\right)\leq o-c\left(o,\theta_{\text{2}}\right)<o-c(o-m,\theta_{\text{2}})$ holds for large m. Therefore, when m is large, it is not more beneficial for the purchaser if supplier 2 wins the contract rather than supplier 1. This is contrary to **Proposition 1(II)** because the extended model introduces δ>0, i.e., the purchaser detects origin falsification with probability δ. If supplier 2 wins, detection occurs with probability δ, leading the purchaser to terminate the contract and procure from an alternative channel at a higher price. This expected cost offsets the advantage of supplier 2 winning, so even when m is large, supplier 2 does not become more beneficial than supplier 1. Then, when E(ε) is large (as in **Proposition 1(I)**), supplier 2 winning leads to lower expected payoff for the purchaser, because a larger E(ε) implies greater utility losses (E(Δo)) from origin falsification. Supplier 2 is always inferior to supplier 1 for the purchaser, which aligns with the conclusion of **Proposition 1(I)**.

Second, we consider the scoring auction. In equilibrium, if supplier 1 wins the contract, his payoff is still $\pi_{\text{1}}=\lambda o_{\text{1}}\left(\lambda \right)-{\overset{―}{s}}_{\text{2}}\left(\lambda \right)-c\left(o_{\text{1}}\left(\lambda \right),\theta_{\text{1}}\right)=\overset{S}{\underset{T}{R}}-\overset{S}{\underset{O}{R}}$. If supplier 2 wins the contract, his payoff is $\pi_{\text{2}}=\lambda o_{\text{2}}\left(\lambda \right)+\lambda m-{\overset{―}{s}}_{\text{1}}\left(\lambda \right)-c\left(o_{\text{2}}\left(\lambda \right),\theta_{\text{2}}\right)=\overset{S}{\underset{O}{R}}-\overset{S}{\underset{T}{R}}$ if undetected, and 0 if detected. In the latter case, the purchaser will procure from an alternative channel at a price no lower than the quoted price of supplier 1. Hence, the purchaser’s utility when falsification is detected is $o_{\text{1}}\left(\lambda \right)-c\left(o_{\text{1}}\left(\lambda \right),\theta_{\text{1}}\right)-\overset{S}{\underset{T}{R}}$. Then, the purchaser’s expected utility can be written as:

$\begin{array}{c} E\left(\overset{S}{\underset{\text{2}}{U}}\left(\lambda \right)\right)=(1-\delta )\cdot \left(\text{utility without detection}\right)+\delta \cdot \left(\text{utility with detection}\right)\\ =(1-\delta )\left(o_{\text{2}}\left(\lambda \right)-c(o_{\text{2}}\left(\lambda \right),\theta_{\text{2}})+\overset{S}{\underset{T}{R}}-\overset{S}{\underset{O}{R}}-\lambda mE\left(\varepsilon \right)\right)+\delta \left(o_{\text{1}}\left(\lambda \right)-c(o_{\text{1}}\left(\lambda \right),\theta_{\text{1}})-\overset{S}{\underset{T}{R}}\right) \end{array}$. Supplier 2’s expected payoff is $E\left(\pi_{\text{2}}\right)=(1-\delta )(\overset{S}{\underset{O}{R}}-\overset{S}{\underset{T}{R}})$.

Then, if supplier 1 wins the contract, we have that


$$\overset{S}{\underset{\text{1}}{U}}\left(\lambda \right)=o_{\text{1}}\left(\lambda \right)-p_{\text{1}}=o_{\text{1}}\left(\lambda \right)-c\left(o_{\text{1}}\left(\lambda \right),\theta_{\text{1}}\right)-\overset{S}{\underset{T}{R}}+\overset{S}{\underset{O}{R}}$$ (11)

If supplier 2 wins the contract, we have that


$$E\left(\overset{S}{\underset{\text{2}}{U}}\left(\lambda \right)\right)=(1-\delta )\left(o_{\text{2}}\left(\lambda \right)-c(o_{\text{2}}\left(\lambda \right),\theta_{\text{2}})+\overset{S}{\underset{T}{R}}-\overset{S}{\underset{O}{R}}-\lambda mE\left(\varepsilon \right)\right)+\delta \left(o_{\text{1}}\left(\lambda \right)-c(o_{\text{1}}\left(\lambda \right),\theta_{\text{1}})-\overset{S}{\underset{T}{R}}\right)$$ (12)

Similar to the price-only auction, the extended model with δ>0 also overturns the result in **Proposition 2(II)**. When supplier 2 wins, the risk of detection (δ) and subsequently costly alternative sourcing creates an expected cost that counteracts any advantage from a large m. Thus, when m is sufficiently large, supplier 2 does not become more beneficial than supplier 1. Meanwhile, when E(ε) is large (as in **Proposition 2(I)**), supplier 2’s winning leads to a lower expected payoff for the purchaser, consistent with the price-only auction case. Hence, in the scoring auction as well, supplier 2 is always inferior to supplier 1 for the purchaser.

In summary, we find that as long as the detection rate δ>0, the purchaser’s decision differs from that in the previous sections. For a sufficiently large m, supplier 2 winning the auction does not lead to a superior outcome under either auction format. This also illustrates the importance of investing in origin detection technology for seafood products.

**Fig 6:**
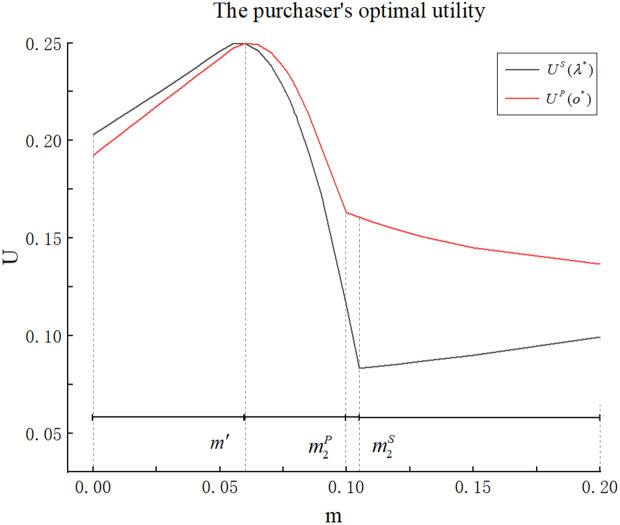
Purchaser’s optimal utility under two auction formats across varying detection rates.

Finally, we compare the two auction formats in the presence of origin detection through numerical studies. For consistency, we use the same parameter values as in Fig 5. In addition we adopt the exponential function $\delta \left(m\right)=1-e^{-\gamma m}$ to model the detection rate. This functional form captures the intuition that as the extent of fraud increases, it becomes increasingly easier to detect, that is, minor falsifications may escape scrutiny, but more egregious deviations are progressively more likely to be uncovered. Specifically, we set γ=5, which is calibrated by setting the detection probability at the upper bound of m (beyond which trade ceases) to a reasonably high level, reflecting effective monitoring. In Fig 6, we compare the purchaser’s optimal utility under the two auction formats across varying detection rates.

Different from the conclusions in the absence of the origin detection, the superiority of the two auction formats now exhibits only two distinct intervals depending on the magnitude of the falsification level. As shown in Fig 6, once the origin falsification level m exceeds $m^{\prime }$, the price-only auction P consistently dominates the scoring auction S for the purchaser and otherwise, the scoring auction dominates. This result differs from the previous findings because the introduction of a positive detection rate δ>0 fundamentally changes the purchaser’s preference. With detection, when supplier 2 wins, the purchaser faces a positive probability of uncovering the falsification, leading to contract termination and costly alternative sourcing. This expected cost grows with m, as δ increases in m. Therefore, for sufficiently large m, the purchaser no longer finds it optimal to let supplier 2 win, unlike in the no-detection case where the scoring auction dominated in this region. Instead, the purchaser consistently prefers supplier 1 to win under both formats. The comparison thus reduces to which format better facilitates procurement from supplier 1. For $m<m^{\prime }$, the scoring auction retains its advantage due to its flexibility. Once m exceeds $m^{\prime }$, the price-only auction dominates, as its direct control over origin requirements, combined with the deterrent effect of detection, allows the purchaser to secure supplier 1 at a lower origin standard and higher utility. Hence, only two intervals remain. This numerical finding suggests that introducing the origin detection tends to simplify the purchaser’s decision-making logic. In our numerical experiments, complex multi-regime structure found in the no-detection case simplifies to a decision based primarily on a single threshold of the falsification level.

## 8. Conclusion

This study conducts a comparative study between price-only auctions and scoring auctions in seafood procurement, considering the impact of origin falsification. Origin falsification allows dishonest suppliers to exaggerate their origin level, granting them an “origin falsification rent”. When this rent outweighs the technological rent, the dishonest suppliers are more likely to win the contract. Adjustments to the origin level requirement (or origin weight) and the level of consumers’ preference for origin influence the disparity between these two rents, thereby affecting both the auction outcome and the purchaser’s optimal utility. Consequently, purchasers must adjust specific procurement auction settings to balance the trade-off between origin falsification and procurement costs. The findings are as follows:

(I) To deter origin falsification, purchasers should enforce stricter origin requirements in price-only procurement auctions (scoring procurement auctions) by increasing the minimum origin level (origin weight).
(II) When origin falsification is serious, procuring seafood from a lower origin level may actually benefit purchasers, especially when consumers show little preference for origin.
(III) Price-only auctions dominate scoring auctions for purchasers in specific scenarios, with the advantage becoming particularly pronounced when consumers exhibit strong origin preferences. This dominance is further strengthened in the presence of origin detection, especially when the extent of falsification is large.

Future research could further investigate the variability in consumers’ preferences for seafood procurement. While our study assumes that consumers’ preferences for origin remain stable, the preceding discussion highlights the potential biases introduced by advertising and market perception. Cantillo et al. [45] suggest that consumers’ preferences are not fixed but rather influenced by factors such as knowledge, awareness of farming practices, and environmental concerns. This variability may lead to misinterpretation of origin-based values, affecting both auction outcomes and procurement strategies. Therefore, future studies should examine how dynamic consumers’ preferences interact with procurement mechanisms and whether adjustments to the minimum origin level (or origin weight) can enhance purchasing efficiency under real-world conditions.

## Supporting information

S1 Appendix Detailed derivations and proofs. (DOCX)

S1 File Detailed data for numerical experiments. (XLSX)
