# Supplementary material for: Seafood procurement auctions in the presence of origin falsification: Price-only auction versus scoring auction
Source: PLoS One. 2026 May 14;21(5):e0348582. doi: 10.1371/journal.pone.0348582 (PMC13175382; doi:10.1371/journal.pone.0348582)
Supplement: S1 Appendix — (DOCX) [file pone.0348582.s001.docx]

**Appendix**

**A.1 Proof of Lemma 1**

We can easily prove the existence of $\hat{o}$ by the single-crossing property. $\hat{o}$ is determined by $c(\hat{o}-m,\theta_{2})-c(\hat{o},\theta_{1})=0$. According to the implicit function theorem,

$\frac{d\hat{o}}{dm}=\frac{c_{o}(\hat{o}-m,\theta_{2})}{c_{o}(\hat{o}-m,\theta_{2})-c_{o}(\hat{o},\theta_{1})}>0$, $\frac{d^{2}\hat{o}}{dm^{2}}=\frac{c_{oo}(\hat{o}-m,\theta_{2})c_{o}(\hat{o},\theta_{1})}{(c_{o}(\hat{o}-m,\theta_{2})-c_{o}(\hat{o},\theta_{1}))^{2}}>0$. Hence, $\hat{o}$ is increasing and convex with $m$. *Q.E.D.*

**A.2 Proof of Proposition 1**

(1-1) According to the setup of the cost function, there is a unique maximum for (1) and (2), denoted as, $o_{1}\equiv\mathrm{argmax}_{o\in[0,\infty)}(1)$ and $o_{2}\equiv\mathrm{argmax}_{o\in[0,\infty)}(2)$ respectively.

(1-2) For any $o\in[0,\hat{o}]$, $(1)>(2)$, because

$(1)-(2)=c(o,\theta_{1})-c(o-m,\theta_{2})+m(1+E(\varepsilon))=\underbrace{c(o,\theta_{1})-c(o-m,\theta_{2})}_{\geq0\text{ by lemma 1}}+m(1+E(\varepsilon))>0$.

(1-3) Compare $o_{1}$ and $\hat{o}$. Because $o_{1}$ is determined by $1-c_{o}(o-m,\theta_{2})=0$(the first derivative of (1)), By the implicit function theorem， $\frac{do_{1}}{dm}=-\frac{c_{oo}(o-m,\theta_{2})}{-c_{oo}(o-m,\theta_{2})}=1$. Hence, $o_{1}$ increases linearly in $m$. By Lemma 1, $\hat{o}$ is increasing and convex with $m$. Therefore, there exists a unique $m_{1}^{P}$ such that for $m<m_{1}^{P}$, $\hat{o}<o_{1}$; for $m>m_{1}^{P}$, $\hat{o}>o_{1}$; and $\hat{o}=o_{1}$ when $m=m_{1}^{P}$.

(1-4) We first consider when $m<m_{1}^{P}$, $\hat{o}<o_{1}$. By (1-2), for all $o\in[0,\hat{o}]$, $(2)<(1)<U_{1}^{P}(\hat{o})$. And$U_{1}^{P}(\hat{o})<U_{1}^{P}(o_{1})$. Therefore, the maximum is at $o_{1}$.

(1-5) When $m>m_{1}^{P}$, $\hat{o}>o_{1}$. Even if $o_{1}$ is selected, supplier 1 cannot strictly outbid the competitor. Therefore, $o^{*}$ is either at $o_{2}$ or $\hat{o}$. By (1-2), $U_{2}^{P}(\hat{o})<U_{1}^{P}(\hat{o})$. Thus, $U^{P}$ exhibits an upward jump at the point of discontinuity. Because $U_{1}^{P}(o)$ is decreasing on $[o_{1},\infty)$ and $\lim_{o\to\infty} U_{1}^{P}(o)=-\infty$. Hence, there exists a unique such that . And because $o_{2}$ does not depend on $m$ and $\hat{o}$ increases with $m$, there exists a unique $m_{2}^{P}\in(m_{1}^{P},\infty)$ that equates $\hat{o}$ and . For $m_{1}^{P}<m<m_{2}^{P}$, ,$U_{2}^{P}(o_{2})<U_{1}^{P}(\hat{o})$, so $o^{*}=\hat{o};$ for then $o^{*}=o_{2}$. If $E(\varepsilon)$ is large enough so that $U_{2}^{P}(o_{2})=o_{2}-c(o_{2},\theta_{1})-m(1+E(\varepsilon))\leq U_{1}^{P}(\hat{o})=\hat{o}-c(\hat{o}-m,\theta_{2})$, then for $m>m_{1}^{P}$, we always choose $o^{*}=\hat{o}$. i.e., . *Q.E.D.*

**A.3 Proof of Lemma 2**

$R_{T}^{S}\equiv\lambda o_{1}(\lambda)-c(o_{1}(\lambda),\theta_{1})-(\lambda o_{2}(\lambda)-c(o_{2}(\lambda),\theta_{2}))$,

$\frac{dR_{T}^{S}}{d\lambda}=o_{1}(\lambda)-o_{2}(\lambda)+o'_{1}(\lambda)[\lambda-c_{o}(o,\theta_{1})]-o'_{2}(\lambda)[\lambda-c_{o}(o,\theta_{2})]=o_{1}(\lambda)-o_{2}(\lambda)>0$. Because

$o_{i}(\lambda)=\mathrm{argmax}\{\lambda o-c(o,\theta_{i})\}$ satisfies first-order condition $H\equiv\lambda-c_{o}(o,\theta_{i})=0$(2-1) By the implicit function theorem, $o'_{i}(\lambda)=-\frac{\partial H/\partial\lambda}{\partial H/\partial o}=1/c_{oo}>0$. Because $c_{oo\theta}>0,c_{oo}(o,\theta_{1})<c_{oo}(o,\theta_{2})$ and thus, $\frac{d^{2}R_{T}^{S}}{d\lambda^{2}}=o'_{1}(\lambda)-o'_{2}(\lambda)=\frac{1}{c_{oo}(o,\theta_{1})}-\frac{1}{c_{oo}(o,\theta_{2})}>0$(2-2). $U_{SB}^{S}=o_{1}(\lambda)-c(o_{1}(\lambda),\theta_{1})-R_{T}^{S}$,

$$\begin{matrix} \frac{dU_{SB}^{S}}{d\lambda}=(1-\lambda)o'_{1}(\lambda)-o_{1}(\lambda)-o_{1}(\lambda)+\lambda o'_{2}(\lambda)+o_{2}(\lambda)-c_{o}(o_{2}(\lambda),\theta_{2})o'_{2}(\lambda) \\ =(1-\lambda)o'_{1}(\lambda)-o_{1}(\lambda)+o_{2}(\lambda)+o'_{2}(\lambda)\underbrace{[\lambda-c_{o}(o_{2}(\lambda),\theta_{2})]}_{=0\text{ by (2-1)}}=(1-\lambda)o'_{1}(\lambda)-o_{1}(\lambda)+o_{2}(\lambda) \end{matrix}$$

$o''_{i}(\lambda)=\frac{d}{d\lambda}(\frac{1}{c_{oo}(o_{i}(\lambda),\theta)})=-\frac{c_{ooo}}{(c_{oo})^{3}}$, , , because

$$\frac{d^{2}U_{SB}^{S}}{d\lambda^{2}}=(1-\lambda)o''_{1}(\lambda)-o'_{1}(\lambda)-o'_{1}(\lambda)+o'_{2}(\lambda)<(1-\lambda)o''_{1}(\lambda)-o'_{1}(\lambda)=-(1-\lambda)\frac{c_{ooo}}{(c_{oo})^{3}}-\frac{1}{c_{oo}}<0$$

$\Leftrightarrow c_{ooo}>-\frac{c_{oo}^{2}}{1-\lambda}$ for all $\lambda\in[0,1]$. . Therefore, there exists a unique maximum $\lambda_{SB}\in(0,1)$ that satisfies . $\lambda_{FB}=\max_{\lambda}\{o_{1}(\lambda)-c(o_{1}(\lambda),\theta_{1})\}=1$, because $o'_{1}(\lambda)-c_{o}(o_{1}(\lambda),\theta_{1})o'_{1}(\lambda)=0\Rightarrow(1-\lambda)o'_{1}(\lambda)=0$. *Q.E.D.*

**A.4 Proof of Lemma 3**

Denote $\xi(\lambda)=\bar{s}_{1}(\lambda)-\bar{s}_{2}(\lambda)=\lambda o_{1}(\lambda)-c(o_{1}(\lambda),\theta_{1})-\lambda o_{2}(\lambda)+c(o_{2}(\lambda),\theta_{2})-\lambda m$

$\xi(\lambda)'=o_{1}(\lambda)-o_{2}(\lambda)-m$ ,$\xi(\lambda)''=o_{1}(\lambda)'-o_{2}(\lambda)'>0$(by (2-2) in Lemma 2).

Combing the condition that $\xi(0)=0$, $\xi(0)'=-m<0$ and $\xi(\lambda)''>0$, we conclude that there exists unique $\hat{\lambda}>0$ such that $\xi(\lambda)<0$ for $\lambda<\hat{\lambda}$;$\xi(\hat{\lambda})=0$ and $\xi(\lambda)>0$ for $\lambda>\hat{\lambda}$.

$\xi(\hat{\lambda})=\hat{\lambda}o_{1}(\hat{\lambda})-c(o_{1}(\hat{\lambda}),\theta_{1})-\hat{\lambda}o_{2}(\hat{\lambda})+c(o_{2}(\hat{\lambda}),\theta_{2})-\hat{\lambda}m=0$. By the implicit function theorem, $\frac{d\hat{\lambda}}{dm}=\frac{\hat{\lambda}}{o_{1}(\hat{\lambda})-o_{2}(\hat{\lambda})-m}>0$ (because $\xi(\hat{\lambda})'=o_{1}(\hat{\lambda})-o_{2}(\hat{\lambda})-m>0$).

$\frac{d^{2}\hat{\lambda}}{dm^{2}}=\frac{2\hat{\lambda}}{(o_{1}(\hat{\lambda})-o_{2}(\hat{\lambda})-m)^{2}}>0$**.** Thus, $\hat{\lambda}$ is increasing and convex in $m$. *Q.E.D.*

**A.5 Proof of Proposition 2**

(2-3) $U_{1}^{S}(\lambda)=U_{SB}^{S}(\lambda)+\lambda m\text{ }$, $U_{1}^{S}(\lambda)''_{\lambda}=U_{SB}^{S}(\lambda)''_{\lambda}<0$. Therefore, $U_{1}^{S}(\lambda)$ is strictly concave and has a unique maximum $\lambda_{1}\equiv\mathrm{argmax}_{\lambda\in[0,\infty)}U_{1}^{S}(\lambda)$.

(2-4) $U_{2}^{S}(\lambda)$ has a bounded domain, $U_{2}^{S}(0)=0$ and $U_{2}^{S}(\hat{\lambda})=o_{2}(\hat{\lambda})-c(o_{2}(\hat{\lambda}),\theta_{2})-\hat{\lambda}mE(\varepsilon)<\infty$, so there exists $\lambda_{2}\equiv\mathrm{argmax}_{\lambda\in[0,\hat{\lambda}]}U_{2}^{S}(\lambda)$. For $\lambda\in[0,\hat{\lambda}]$, $R_{T}^{S}\leq R_{O}^{S}$, we have

$U_{1}^{S}(\lambda)-U_{2}^{S}(\lambda)=\underbrace{2(R_{o}^{S}-R_{T}^{S})}_{\geq0}+\underbrace{(o_{1}(\lambda)-c_{1}-(o_{2}(\lambda)-c_{2}))}_{>0}+\lambda mE(\varepsilon)>0$.

(2-5) Compares $\lambda_{1}$ and $\hat{\lambda}$. Because $U_{1}^{S}(\lambda)=U_{SB}^{S}(\lambda)+\lambda m\text{ }$, we can show that

, (by the implicit theorem). So $\lambda_{1}$ increases linearly in $m$. By lemma 3, $\hat{\lambda}$ is increasing and convex in $m$. Therefore, there exists a unique $m_{1}^{S}$ such that for $m<m_{1}^{S}$, $\hat{\lambda}<\lambda_{1}$; for $m>m_{1}^{S}$, $\hat{\lambda}>\lambda_{1}$ and $\hat{\lambda}=\lambda_{1}$ when $m=m_{1}^{S}$.

(2-6) When $m<m_{1}^{S}$, $\lambda^{*}=\lambda_{1}$. By (2-4), for all $\lambda\in[0,\hat{\lambda}]$, $U_{2}^{S}(\lambda)<U_{1}^{S}(\lambda)<U_{1}^{S}(\hat{\lambda})<U_{1}^{S}(\lambda_{1})$. Hence, the maximum of (4) is at $\lambda_{1}$.

(2-7) When $m>m_{1}^{S}$, $\hat{\lambda}>\lambda_{1}$, $\lambda^{*}$ is either at $\hat{\lambda}$ or $\lambda_{2}$. By (2-4), for $\lambda_{2}\in[0,\hat{\lambda}]$, $U_{2}^{S}(\lambda_{2})<U_{1}^{S}(\lambda_{2})\leq U_{1}^{S}(\lambda_{1})$. This is because $U_{1}^{S}$ is decreasing on $[\lambda_{1},\infty)$ and $\lim_{\lambda\to\infty} U_{1}^{S}(\lambda)=-\infty$. There exists a unique $\breve{\lambda}\in[\lambda_{1},\infty)$ such that $U_{1}^{S}(\breve{\lambda})=U_{2}^{S}(\lambda_{2})$. If $\hat{\lambda}<\breve{\lambda}$, $U_{2}^{S}(\lambda_{2})<U_{1}^{S}(\hat{\lambda})$, $\lambda^{*}=\hat{\lambda}$. If $\hat{\lambda}>\breve{\lambda}$, $U_{2}^{S}(\lambda_{2})>U_{1}^{S}(\hat{\lambda})$, $\lambda^{*}=\lambda_{2}$. Because $\hat{\lambda}$ increases in $m$, we can find $m_{2}^{S}\in(m_{1}^{S},\infty)$ that equates $\hat{\lambda}$ and $\breve{\lambda}$. For $m_{1}^{S}<m<m_{2}^{S}$, $\hat{\lambda}<\breve{\lambda}$, $\lambda^{*}=\hat{\lambda}$; for $m>m_{2}^{S}$, $\hat{\lambda}>\breve{\lambda}$, $\lambda^{*}=\lambda_{2}$. If $E(\varepsilon)$ is large enough so that

$U_{2}^{S}(\lambda_{2})=o_{2}(\lambda_{2})-c(o_{2}(\lambda_{2}),\theta_{2})+\lambda_{2}o_{1}(\lambda_{2})-c(o_{1}(\lambda_{2}),\theta_{1})-(\lambda_{2}o_{2}(\lambda_{2})-c(o_{2}(\lambda_{2}),\theta_{2}))-\lambda_{2}m-\lambda_{2}mE(\varepsilon)$ $\leq U_{1}^{S}(\hat{\lambda})=o_{1}(\hat{\lambda})-c(o_{1}(\hat{\lambda}),\theta_{1})$, then for $m>m_{1}^{S}$, we always choose $\lambda^{*}=\hat{\lambda}$. i.e.,

. *Q.E.D.*
